# Correction of Mild-to-Moderate Constricted Ear Abnormality Using Mustardé Suture, Cartilage Onlay Graft, and Transposition Flap: A Case Report

**DOI:** 10.1055/a-2073-4083

**Published:** 2023-08-02

**Authors:** Ha Jong Nam, Syeo Young Wee

**Affiliations:** 1 Department of Plastic and Reconstructive Surgery, Soonchunhyang University Gumi Hospital, Gumi, Republic of Korea

**Keywords:** ears prominent and constricted, external ear cartilage, surgical flaps

## Abstract

Constricted ear has a prevalence of 5.2 to 10% among ear abnormalities, and various surgical methods are suggested for treatment. We introduce a case of a constricted ear treated with a simple method using a novel concept cartilage graft and transposition flap, along with the well-known Mustardé suture, which is used for pediatric patients with mild to moderate constricted ears of Tanzer classification type IIA. A 10-year-old female patient visited the hospital complaining of an abnormality in the congenital right ear. Surgical approach was planned under the diagnosis of Tanzer classification type IIA constricted right ear. Posterior helix onlay graft and perichondrocutaneous transposition flap using excessive helical cartilage were performed along with the Mustardé suture. In the immediate postoperative period, ear contour was improved, and it was well-maintained without recurrence until 6 months' follow-up. In conclusion, the combination of Mustardé suture, and cartilage onlay graft and perichondrocutaneous transposition flap in the mild to moderate constricted ear would be a useful surgical option, producing aesthetically good results in a simple and effective method.

## Introduction

Constricted ear, also known as lop ear or cup ear, is a congenital ear abnormality. The etiology is related to problems in the process of spreading the upper helix over the antihelix at 3 to 4 months of gestational age. 1 2 3 Hooding, which is the downward morphological folding of the upper portion of the helical rim, causes deficiencies in the scapha, superior crus, and triangular fossa. 3 4 In severe cases, decreased vertical height of the ear and lower ear position can be observed due to the deepened conchal fossa, anterior projection of the upper portion of the ear, and vertical compression of the triangular fossa of the scapha. 2

Constricted ears are most often corrected by molding a conforming splint in neonatal period. 5 6 Early initiation of the ear molding technique results in higher success rates and shorter overall treatment periods. 7 It is recommended to perform the procedure between 1 week to 3 months of age. 8 If performed before 2 weeks of age, a satisfactory correction success rate of up to 91.2% has been reported. 9 However, after the loss of flexibility of the cartilage, these methods are not effective; hence, surgery is the main treatment. 10 In the literature, the patient group that underwent surgical treatment for constricted ear showed a frequency of 5.2 to 10% of ear abnormalities. In general, many surgeons consider that before entering elementary school, between the ages of 3 and 6 years is the most appropriate timing for correction of constricted ears. 2 4 11

Various methods of flap and cartilage refashioning are used for surgical treatment of constricted ears, but it is controversial as to which method has the most efficient and best results, and numerous modifications are made according to individual clinical conditions. In particular, for the surgical correction of mild to moderate constricted ears, good results are difficult to obtain with a solitary method; hence, there have been several reports of using a combination of various methods. 10 Therefore, we present a simpler surgical method using a new concept cartilage graft and transposition flap along with the well-known Mustardé suture for pediatric patients with mild to moderate constricted ears of Tanzer classification type IIA.

## Case

A 10-year-old female patient visited the hospital complaining of a right ear abnormality, which was observed since birth. The patient had no history of other treatments, including molding for the affected ear. Due to the shape of the ear, it was inconvenient to fix the upper ear when wearing a mask, and the patient preferred morphological normalization.

On physical examination, the right ear had a congenital morphological abnormality. At the time of the visit, the ear shape showed hooding of the upper lateral portion of the helical rim (Darwin's tubercle area), and flattening of the superior and inferior crura and body of the antihelix was observed ( Fig. 1 ). The size of the entire ear was 5.5 cm in length and 2.7 cm in width, and the affected side had a height reduction of approximately 0.4 cm compared with the contralateral side. The auriculocephalic angle was 35 degrees, slightly increased compared with 32.5 degrees on the contralateral side. The position of the ear was observed similar to that of the contralateral side when it was located at a distance of 6.8 cm from the lateral canthus of the eye. No additional otolaryngological problems were observed, such as hearing impairment. We planned a surgical approach under the diagnosis of constricted ear Tanzer classification type IIA for morphological deformity of the right ear. The contralateral ear was also classified as a Tanzer classification type I, however, the patient expressed her reluctance to correct for mild deformity. As a result, our surgical intervention was limited to addressing only the right side.

**Fig. 1:**
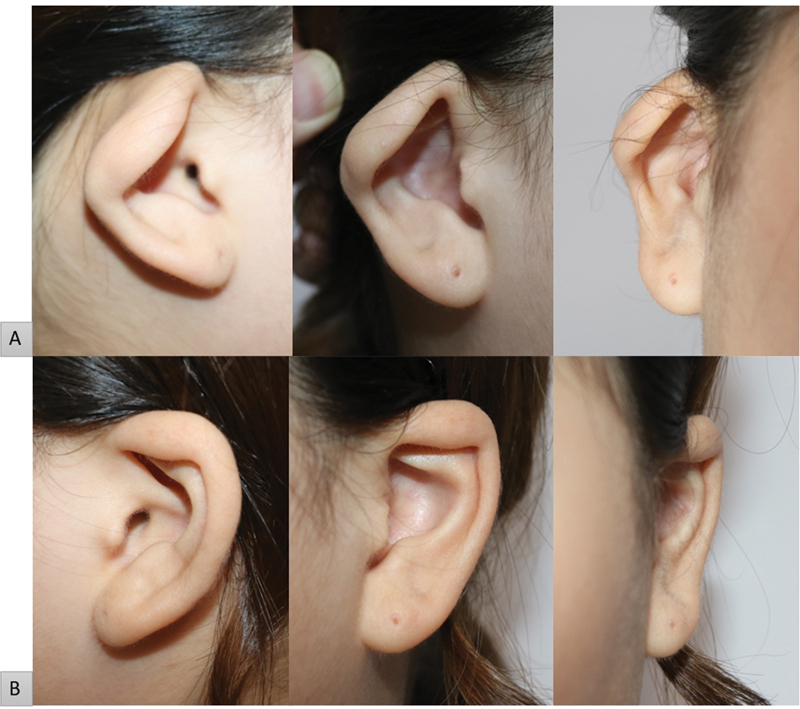
Preoperative photographic findings. ( **A** ) Affected side. Hooding was observed in the upper lateral portion (Darwin's tubercle area) of the helical rim, and flattening of the superior and inferior crura and body of the antihelix was observed. ( **B** ) Contralateral side for comparison.

First, the incision line of the Z-shape was drawn with the tip at an angle of 40 degrees to cover the upper two-thirds of the posterior side of the right ear ( Fig. 2A ). Subperichondrial dissection was performed under skin incision to expose from the root of the helical cartilage to the anterior margin of the center portion. There was thin and wide excessive cartilage of the helix inside the part with apparent hooding.

**Fig. 2:**
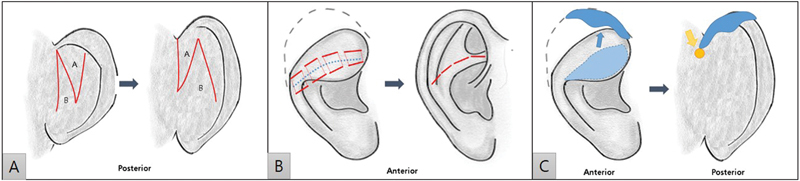
Illustration of surgical procedure. ( **A** ) Z-shaped incision line drawn on the posterior surface of the ear. It was transposed between A and B. ( **B** ) Mustardé suture was performed on the flattened antihelix. ( **C** ) Cartilage harvest of lop ear and graft to upper posterior portion in onlay fashion were done (blue color). Helical root was fixed to the deep temporal area for additional correction (yellow color).

On the posterior surface of the antihelix showing flattening, the area to create a square antihelical crura was designed, and after designing the area for Mustardé suture, a horizontal mattress Mustardé suture was performed at four points ( Figs. 2B and 3 ). 10 Subsequently, the excessive cartilage of the helical upper lateral portion was compared with that of the opposite ear and marked, and the whole cartilage was excised as one block. The size of the excised cartilage was confirmed to be 3.7 cm × 0.7 cm. Onlay grafting was performed on the cartilage on the upper posterior side of the helix so that the concave side of the cartilage was engaged with the posterior side. After fixation was performed on the lateral end, fixation was performed on the deep temporal portion, which is the inner most part of the helical root, at the medial end ( Figs. 2C and 4 ). After suture fixation and grafting, the hooding of the upper lateral portion of the helical rim and the flattening of crura and body of the antihelix were corrected ( Fig. 5 ). Skin closure was performed by wrapping the entire construction of ear cartilage through transposition on the perichondrocutaneous flap of a previous design **Z** -shape ( Fig. 2A ).

**Fig. 3:**
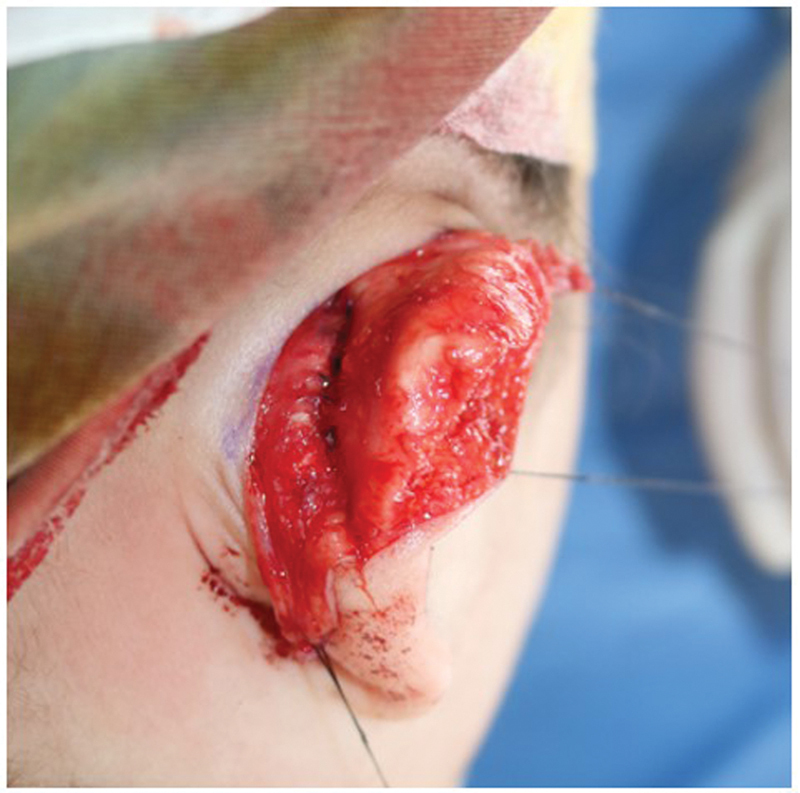
Intraoperative photograph. Mustardé suture was applied.

**Fig. 4:**
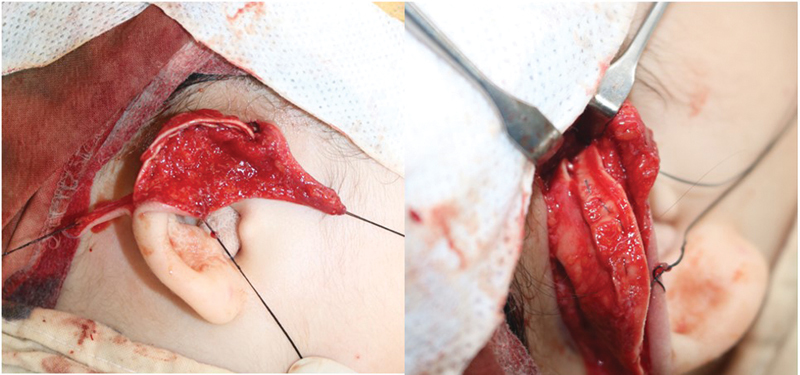
Excessive cartilage was resected and onlay grafting was performed for reinforcement of helix.

**Fig. 5:**
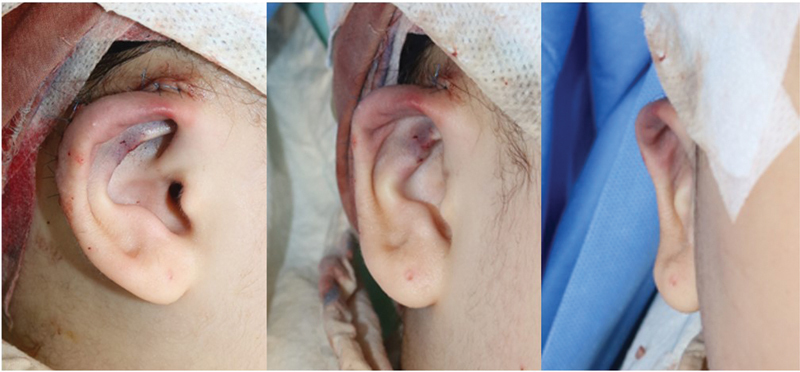
Immediate postoperative photographs. The hooding of the upper lateral portion of the helical rim and the flattening of crura and body of the antihelix were corrected.

Postoperatively for 1 week, dressing was performed by applying molding using Vaseline gauze to the anterior and posterior directions of the ear. After surgery, wound healing was achieved without hematoma or infection. The shape of the ear was successfully maintained without any additional signs of recurrence or complications, except for a minimal scar appearance on the posterior surface of the ear observed 6 months after the surgery ( Fig. 6 ).

**Fig. 6:**
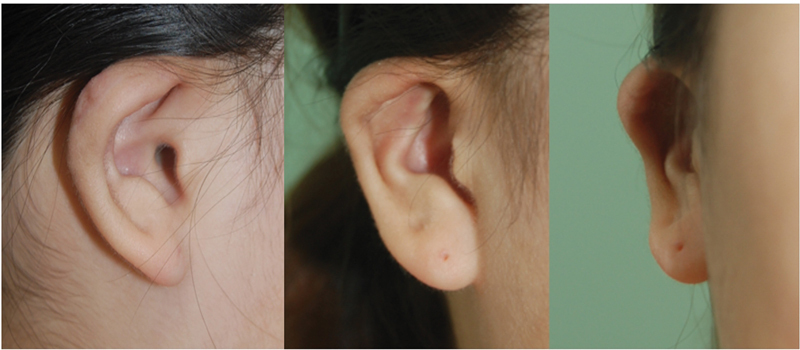
Photograph taken 6 months after operation. The shape of the ear was maintained, without recurrence or complications.

## Discussion

Tanzer classification type IIA is defined as moderate compression of the helix and scapha, and distinguished from type IIB in that loss of vertical height is observed and with little need for supplemental skin. 1 A wide variety of surgical methods have been proposed for the treatment of type IIA. To improve the shape of the ear, the cartilage is straightened by inserting an incision in the characteristic finding, which is the bent helix shape or the lop ear. Methods such as Barsky's, Ragnell's, and Stephenson's, and the Margrave technique have been introduced. 12 13 14 15 Furthermore, various methods using flaps have been recently introduced, with the V-Y advancement flap being commonly utilized in multiple ways. This flap is inserted into the preauricular area to reinforce vertical height, and into the posterior surface to expand the skin envelope after cartilage reconstruction and to expand the angulated cartilage of the upper helix in the helical root. 16 17 18 Mustardé suture is also one of the treatments for the constricted ear correction to create an antihelical fold. 10

In mild to moderate constricted ears, it is difficult to maintain the shape of the antihelix crus with only the Mustardé suture, and to reinforce this, a method for securing the crus shape through reinforcement using concha cartilage or free-floating costal cartilage has been introduced. 19 20 However, these methods require additional procedures for harvesting of the concha and costal cartilage. On the other hand, our method achieves morphological correction in a simpler way that does not require an additional procedure for additional cartilage harvesting by using excessive cartilage that forms helix hooding. Our method reinforces the curvature of the helix through onlay graft on the posterior side to reduce the concavity of the helix, which is distinguished from the existing cartilage graft. Through this, the auriculocephalic angle of the upper portion of the ear could be made sharper. In addition, the method of reinforcing with free cartilage not only made it possible to place the cartilage more accurately at the position to be reinforced, but also made a clear correction effect by fixing the end of the cartilage to the deep temporal area ( Fig. 2C ).

For excessive posterior side skin, the upper flap is positioned as the auriculocephalic sulcus after transposition by utilizing Z-shape transposition flaps for initial incision and skin closure. This technique corrects the curvature of the helix through tightening of the posterior skin after cartilage reposition. Furthermore, the space of the upper portion of the helix, which was secured through the transposition flap, was able to secure and reinforce the fixation of the cartilage end through the onlay graft on the posterior side in the temporal area.

In conclusion, for a mild to moderate constricted ear case of Tanzer classification IIA, we performed a simple and effective method that combined the posterior helix onlay graft and perichondrocutaneous transposition flap using excessive helical cartilage along with the existing Mustardé suture. We concluded that this method was able to produce good results and can be used for various cases of constricted ear surgery.
